# Age-related cerebral small vessel disease and inflammaging

**DOI:** 10.1038/s41419-020-03137-x

**Published:** 2020-10-30

**Authors:** Tiemei Li, Yinong Huang, Wei Cai, Xiaodong Chen, Xuejiao Men, Tingting Lu, Aiming Wu, Zhengqi Lu

**Affiliations:** 1 Department of Neurology, the Third Affiliated Hospital of Sun Yat-sen University, Guangzhou, Guangdong 510630 China; 2 Mental and Neurological Diseases Research Center, the Third Affiliated Hospital of Sun Yat-sen University, Guangzhou, Guangdong 510630 China; 3 Department of Endocrinology and Diabetes Center, the First Affiliated Hospital of Sun Yat-sen University, Guangzhou, Guangdong 510080 China

**Keywords:** Neuroimmunology, Cerebrovascular disorders

## Abstract

The continued increase in global life expectancy predicts a rising prevalence of age-related cerebral small vessel diseases (CSVD), which requires a better understanding of the underlying molecular mechanisms. In recent years, the concept of “inflammaging” has attracted increasing attention. It refers to the chronic sterile low-grade inflammation in elderly organisms and is involved in the development of a variety of age-related chronic diseases. Inflammaging is a long-term result of chronic physiological stimulation of the immune system, and various cellular and molecular mechanisms (e.g., cellular senescence, immunosenescence, mitochondrial dysfunction, defective autophagy, metaflammation, gut microbiota dysbiosis) are involved. With the deepening understanding of the etiological basis of age-related CSVD, inflammaging is considered to play an important role in its occurrence and development. One of the most critical pathophysiological mechanisms of CSVD is endothelium dysfunction and subsequent blood-brain barrier (BBB) leakage, which gives a clue in the identification of the disease by detecting circulating biological markers of BBB disruption. The regional analysis showed blood markers of vascular inflammation are often associated with deep perforating arteriopathy (DPA), while blood markers of systemic inflammation appear to be associated with cerebral amyloid angiopathy (CAA). Here, we discuss recent findings in the pathophysiology of inflammaging and their effects on the development of age-related CSVD. Furthermore, we speculate the inflammaging as a potential target for future therapeutic interventions to delay or prevent the progression of the age-related CSVD.

## Facts

- The latest cellular and molecular mechanisms of inflammaging include cellular senescence, immunosenescence, mitochondrial dysfunction, defective autophagy, metaflammation and gut microbiota dysbiosis.
- The relationship between inflammaging and age-related CSVD is explored from the two following aspects: vascular inflammation and DPA; systemic inflammation and CAA.
- The contributions of inflammaging in age-related CSVD are potential target for future therapeutic interventions.

## Open questions

- Does inflammaging play a role in explaining the pathophysiological interactions between DPA and CAA?
- Future research should focus on the co-relationship between gut microbiota and inflammaging in age-related CSVD patients.
- Clinical studies are needed to assess the effectiveness of inflammaging-targeted interventions for age-related CSVD.

## Introduction

Cerebral small vessel disease (CSVD) is defined as a group of CSVD, in which cerebral small arteries, arterioles, capillaries, venules, small veins are attributed to atherosclerotic, genetic, idiopathic, infectious, immune-mediated or other secondary etiologies^1^. The age-related CSVD includes deep perforating arteriopathy (DPA) and β-amyloid (Aβ) related cerebral amyloid angiopathy (CAA). Age-related CSVD is most prevalent among the elderly and contributes to high global disease burden of stroke and vascular dementia. Advanced age and hypertension are the most accepted epidemiologic risk factors^2^. An increased incidence of age-related CSVD accompanies the steady worldwide increase in life expectancy, affects about 5% of people aged 50 years and almost everyone older than 90 years, leading to a huge social and economic burden^1^.

**Fig. 1:**
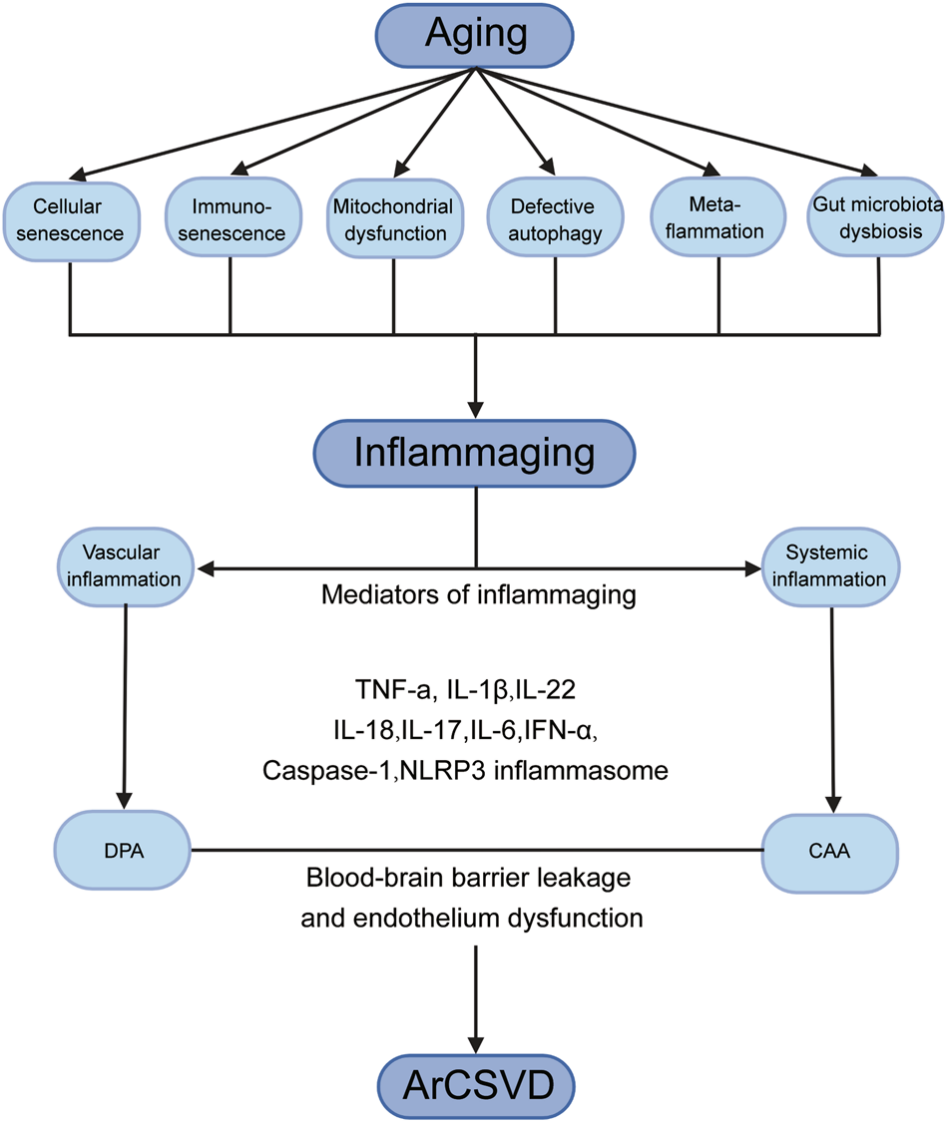
The deep interactions between aging, inflammaging, and age-related CSVD. As aging, several cellular and molecular mechanisms lead to chronic inappropriate activation of the immune system. This complex interaction between genetic susceptibility and risk stimuli (both exogenous and endogenous) contributes to the continuous activation of a limited range of confounding sensors which triggers inflammaging (upper part of the box). The resulting synthesis and release of different inflammatory mediators are related to the common pathophysiological mechanisms of age-related diseases. For age-related CSVD, regional analyses showed that blood markers of vascular inflammation were associated with deep perforating arteriopathy (DPA), while blood markers of systemic inflammation were associated with cerebral amyloid angiopathy (CAA), both of which were closely related to the critical pathophysiological mechanisms of blood-brain barrier leakage and endothelial dysfunction (lower part of the box).

Aging is characterized by a progressive decline in multiple physiological processes, which makes the elderly a highly vulnerable population. Aging is a major risk factor for chronic non-communicable diseases (e.g. diabetes, cancer, cardiovascular diseases, CSVD), among which inflammaging plays an important role^3^. The term inflammaging describes the condition of chronic sterile low-grade inflammation observed in older organisms^4^. Inflammaging is the long-term result of chronic physiological stimulation of the immune system, and possess various cellular and molecular mechanisms, including cellular senescence, immunosenescence, mitochondrial dysfunction, defective autophagy, metaflammation, and gut microbiota dysbiosis^5^ (Fig. 1).

An important pathophysiological mechanism of CSVD is endothelium dysfunction and subsequent blood-brain barrier (BBB) leakage, which gives a clue in the identification of the disease through circulating biological markers^2^. As deepening understanding in the etiological basis of age-related CSVD, inflammaging has been proposed as a candidate factor. Typical markers of inflammation can be classified as systemic inflammatory factors (e.g., C-reactive protein, interleukin-6) or vascular inflammation/endothelial dysfunction (e.g., homocysteine, von Willebrand factor). A previous study indicated a strong correlation between vascular inflammatory markers and CSVD, especially in CSVD patients with stroke^6^. Meanwhile, longitudinal survey illustrated that baseline expression levels of systemic inflammatory factors could predict the severity of the subsequent CSVD. More importantly, the regional analysis showed that systemic and vascular inflammation was associated with two different subtypes of CSVD (CAA and DPA), respectively. To be specific, markers of vascular inflammation tended to be associated with DPA (e.g., basal ganglia), whereas systemic inflammation seemed to be associated with CAA (e.g. centrum semiovale)^6^ (Fig. 1).

This review focuses on the latest research of inflammaging, systemic inflammatory biomarkers and pathophysiological progression of CSVD, and explores the potential link between inflammaging and age-related CSVD. Besides, we discuss the potential role of inflammaging as a target for future therapeutic interventions to delay or prevent the progression of the age-related CSVD.

## Inflammaging

The term inflammaging was coined in the early 21st century to refer to a persistent sterile, low-grade systemic pro-inflammatory status that occurs during aging process in mammals^4^. Inflammation is a valid defense mechanism to resist harmful substances invading the body and maintain homeostasis. However, the harmful effects of chronic subclinical inflammation on the body might be an essential risk factor for increasing the incidence of degenerative diseases (e.g., osteoporosis) and metabolic diseases (e.g., type 2 diabetes) in the elderly^7^.

**Fig. 2:**
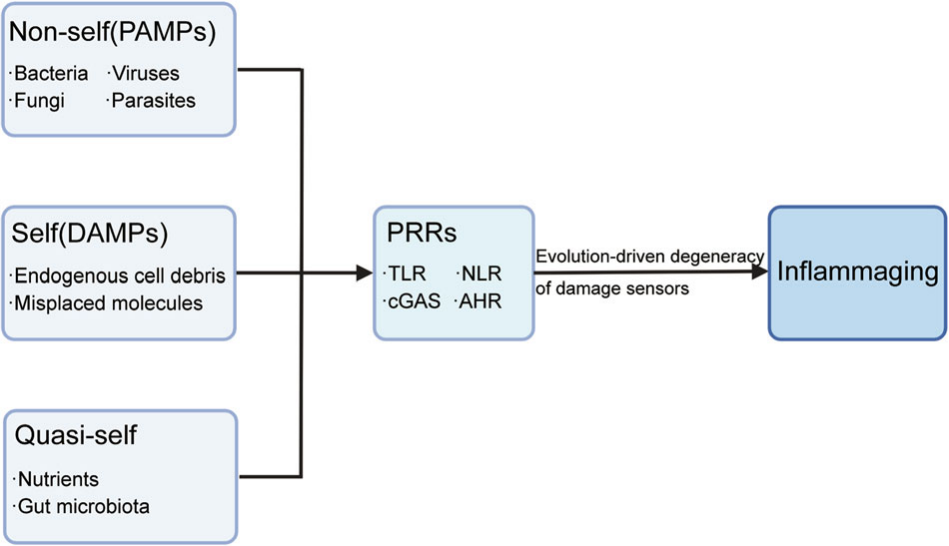
The degeneracy of the damage sensor triggers inflammaging. Exogenous and endogenous danger stimuli interact with pattern recognition receptors (PRRs) expressed on the cell surface and in the cytoplasm. Danger molecules can be non-self (pathogen-associated molecular patterns, PAMPs), self (damage-associated molecular patterns, DAMPs) and quasi-self (nutritional and metabolic products from the gut microbiota). These multitude of stimuli converge on the same evolutionarily selected promiscuous sensors, and trigger inflammaging.

The innate and adaptive immune systems protect our bodies from harmful substances and inappropriate stimuli, which promote the development of inflammaging. These stimuli include pathogens such as bacteria, viruses, fungi and parasites (non-self), endogenous cell debris and misplaced molecules (self), and nutrients and gut microbiota (quasi-self)^8^. A limited number of sensors in the body sense the stimuli, whereas their degeneracy allows them to identify many signals and activate the downstream cascades. Pattern recognition receptors (PRRs), including toll-like receptors (TLRs)^9^, NOD-like receptors, cyclic GMP-AMP synthetase (cGAS), aryl hydrocarbon receptor (AHR), have the degeneracy of biological functions. These receptors can recognize different stimuli produced by non-self molecules including viral and bacterial products (pathogen-associated molecular patterns, PAMPs), self molecules (termed damage-associated molecular patterns, DAMPs) and nutritional and metabolic products from the gut microbiota (which could be considered as quasi-self)^8^. Inflammaging is an unpredicted consequence of the evolution-driven degeneracy of damage sensors^10^ (Fig. 2).

Inflammaging is a situation under which immunity exhibits antagonistic pleiotropy during aging. It involves a variety of different cellular and molecular mechanisms,which are synergistic and mutually promoting^11^ as detailed below.

### Cellular senescence

The leading self-stimuli of inflammaging are the accumulation of damaged macromolecules and cellular debris, which could chronically inhibit the capacity of multiple tissues to surveil and repair damage^12^. Thus, cellular senescence may be the cause of inflammaging. Growing evidence shows that senescent cells (SCs) exert detrimental effects on the tissue micro-environment by generating pathological facilitators. Accordingly, it has been suggested that SCs contribute to the development of inflammaging through the generation of senescence-associated secretory phenotype (SASP), which consists of a variety of soluble factors, such as pro-inflammatory mediators (e.g., IL-6, IL-8) and matrix-degrading molecules^13^. Non-degradable wastes in the body chronically activates innate immunity via self-stimuli of DAMPs, in which macrophages play a central role^14^. Innate immune cells subsequently activate classical pro-inflammatory pathways such as nuclear factor kappa-light-chain enhancer of activated B cells (NF-κB) and signal transducer and activator of transcription (STAT), and secrete a large amount of pro-inflammatory cytokines/chemokines (e.g., IL-6)^15,16^.

In addition to immune cells and tissue cells, tissue-resident stem cells, especially mesenchymal stromal cells (MSCs), are also affected by cellular senescence^17^. Similar to other adult stem cells, MSCs possess a limited capacity of self-renewal. The aging process leads to the age-associated decline in cell number and dysfunction of MSCs^18^. Aged MSCs existing in vivo may participate in the development of inflammaging exogenously through the following mechanisms: a. Aged MSCs produce large amounts of cellular wastes *in vivo*, which act as an important source for DAMPs and exacerbate the onset and development of inflammaging. b. Aged MSCs acquire SASP by producing a robust amount of pro-inflammatory cytokines, including IL-6, IL-8, interferon-gamma (IFN-γ), monocyte chemoattractant protein (MCP)-1, and matrix metalloproteinases (MMP2, TIMP2). c. MSCs possess potent immunomodulatory capacity (such as promoting macrophage polarization from M1 to M2), whereas the aging process compromised these beneficial effects. d. MSC is one of the critical components of the hematopoietic stem cell (HSC) niche, which plays a crucial role in maintaining HSC homeostasis and bone marrow microenvironment. Age-associated mutations hinder niche functions of MSCs and compromise their hematopoietic supportive function in the elderly^19^.

### Immunosenescence

In addition to inflammaging (over-reaction), an important feature of the immune system aging is immunosenescence (insufficiency)^20^. Immunosenescence refers to the destruction of immune organ structure and the dysfunction of the immune response, which leads to a decline in the preservation and enhancement of many immune functions. It is the result of combined actions of innate immunity and adaptive immunity^21^. Although immunosenescence and inflammaging seem to be antipathic phenotypes, there are many overlaps in their mechanisms and progression from the perspective of immune dysfunction^20^.

In the immune system, there is an innate compartment, consisting of neutrophils monocytes/macrophages, natural killer cells, and dendritic cells, and an adaptive compartment, composed of B and T cells^22^. As the first barrier against pathogens, the immunosenescence of the innate immune system is extremely complex and seems to reflect dysregulation, not just dysfunction. In fact, some responses of the innate immune system decrease with age, but the age-related hyperreactivity of innate immunity has also been reported^23^. Immunosenescence of the adaptive immune system is characterized by reduced naive CD4^+^ and CD8^+^ T cell counts, an imbalance of T cell subsets, and a decrease in T cell receptor (TCR) repertoire and signaling. The development and selection of T cells occur in the thymus. Thymus degeneration leads to a decrease in the frequency and number of naïve T cells, an increase in the number of terminally differentiated T cells, and a decrease in the expression of TCR^24^. The numerical reduction in naïve T cells and TCR-reduced repertoire result in T cells dysfucntion^25^. Similarly, there is a decline in B cells and a reduction in antibody production^26^. B cells follow a well-defined developmental process, starting from naïve cells that do not produce specific antibodies isotype, to the establishment of peripheral B cell pools that maintains maturity through self-renewal^27^. The most significant alterations of B cells during aging are loss of naïve B cells, impaired capacity to response to new antigens, reduction of clonal expansion of memory cells, and weakened antibodies function^28^.

### Mitochondrial dysfunction and defective autophagy

Mitochondrial metabolism and autophagy are the two most metabolically active cellular processes and play an important role in regulating the lifespan of organisms^29^. Mitochondria control a large number of cellular processes^30^, not only by controlling the production of ATP, but also by acting as biosynthetic and signaling centers. Autophagy is a cellular process responsible for degrading damaged organelles and protein aggregates. Autophagy degrades several organelles, one of which deserves particular attention is autophagy-mediated mitochondrial degradation called mitophagy^31^.

There is an apparent interconnection between mitochondria and autophagy, both of which promotes the process of inflammaging^32^. The crosstalk of mitochondrial dysfunction and defective autophagy favors the activation of several inflammatory pathways. For example, autophagy defects promote the accumulation of dysfunctional mitochondria, leading to the direct release of large amounts of mitochondrial DNA (mtDNA) into the cytoplasm. MtDNA in the cytoplasm induces the activation of caspase-1 and subsequent production of IL-1β^33,34^. Besides, mitochondria are the signaling hubs^33^ that regulate intracellular calcium pool and reactive oxygen species (ROS) levels, both of which are classical inflammatory mediators^35,36^. Maintenance of mitochondrial and endoplasmic reticulum contact sites regulates leukocyte migration and lymphocyte activation by balancing intracellular calcium pool and regulating autophagy induction^37^. In the oxidation-inflammation theory of aging, it is proposed that accumulated mtDNA mutations could disrupt mitochondrial respiratory chain and leads to excessive mitochondrial ROS (mtROS) production. In turn, this accelerates the emergence of new mutations in mtDNA (leading to cellular senescence) and aggravates the inflammaging process. In addition, aging leads to the further production of ROS and inflammatory mediators, namely oxi-inflamm-aging, forming a vicious cycle^38^.

### Metaflammation and gut microbiota dysbiosis

Metaflammation refers to metabolic inflammation caused by nutrient excess or overnutrition^8^, which exists in chronic age-related metabolic diseases (e.g. type 2 diabetes, obesity). Metaflammation is the most convincing mechanism linking nutritional disorders to inflammaging, indicating that the inflammatory milieu of metabolic cells, tissues and organs is altered due to the high nutrient intake^8^. A pro-inflammatory milieu characterized by high-caloric intake promotes insulin resistance^39,40^. The level of pro-inflammatory cytokines increases during the process of inflammaging, which promotes the infiltration of immune cells in insulin-responsive tissues (e.g., fat and muscle), thereby increase local oxidative stress and inflammatory response, and decrease the expression of insulin receptors^41,42^. Besides, in the post-prandial state, residual chylomicrons and very low-density lipoprotein (VLDL) bind to endothelial cells (ECs) and circulating leukocytes, resulting in acute cell activation and a surge of adhesion molecules, cytokines, and oxidative stress, which ultimately leads to inflammaging^43–45^.

As a cross between metabolism and inflammation, the gut microbiota has a central role in both metaflammation and inflammaging regulation^43^. The human gut microbiota is a highly diverse ecosystem, composed of trillions of bacteria, each part of which has a specific role and respond to a variety of signals from the host. These microbiota alter their own activities to achieve mutualism with the host^46^. Beneficial and potentially pathogenic microorganisms have similar epitopes, gain immunological features through their evolution, and acquire immune tolerance in the host^47–49^. An analysis of the human gut microbiota showed that among Italian individuals (aged 22–109 years), the core population of gut microbiota exhibited a decrease in diversity and relative abundance with aging^50^. In the gut microbiota of the elderly, there is an enrichment of proteobacteria and a decrease in butyrate-producing bacteria, which leads to the decrease of Treg cells, indirect increase of Th17 cells and the generation of pro-inflammatory cytokines including IL-6, IL-8,IL-17^51^. In mice studies, the gut microbiota of aged mice was inoculated into young germ-free mice, causing an inflammatory response with an increased proportion of T helper (Th) cell subsets and increased levels of inflammatory markers such as tumor necrosis factor (TNF). This might be related to the increased inflammatory potential of the gut microbiota of aged mice^52,53^.

Aging and age-related diseases share these basic mechanism pillars, which ultimately converge on inflammation to a large extent. In the aging process, inflammaging promotes the occurrence of age-related diseases. Next, we will further discuss the relationship between inflammaging and age-related CSVD.

## The relationship between inflammaging and age-related CSVD

Inflammaging is increasingly recognized as a risk factor for dementia, stroke, and CSVD^6^. Inflammaging not only works through the aging immune system, but also interacts with traditional cerebrovascular risk factors (e.g. obesity, hypertension, and type 2 diabetes) to exacerbate their harmful effects. In CSVD patients, lesions such as repeated mild stroke lead to BBB leakage, central nervous system antigen release into the peripheral circulation, and lymphocyte infiltration into brain tissue and related cerebral dysfunction^54^. In turn, brain dysfunction may further damage the immune system, forming a vicious circle^55^. Thus, the relationship between inflammaging and the development of age-related CSVD deserves our attention.

Circulating biomarkers of CSVD inflammation were classified as markers of systemic inflammation and markers of vascular inflammation/endothelial dysfunction^6^. Besides, four core MRI features have been identified as imaging markers of CSVD, namely white matter hyperintensities (WMH), lacunae, cerebral microhemorrhage (CMB), and perivascular space enlargement (EPVS)^56^.

We will explore the relationship between inflammaging and age-related CSVD from the following two aspects: vascular inflammation and DPA; systemic inflammation and CAA.

### Vascular inflammation and deep perforator arteriopathy

DPA mainly affects small arteries, veins, arterioles, venules and capillaries (diameter ranging between 100 and 400 microm). These small arteries are derived from deep perforating vessels of large vessels at the base of the brain or penetrating cortical/medullary vessels from superficial medium-sized arteries^57^. The main lesions in the blood supply area of DPA result in WMH in the periventricular region, deep CMB and EPVS in the basal ganglia, etc. For anatomical reasons, the supply vessels in these areas are more likely to narrow due to atherosclerosis, especially in elderly patients with hypertension and diabetes^58–60^. Furthermore, BBB and endothelial dysfunction seem to play important roles in DPA. Jandke et al.^61^ proved that rats with DPA exhibited injured endothelial tight junctions and leakage of proteins deposited surrounding the small vessel walls.

Pro-inflammatory cytokines and inflammatory cells are involved in the formation of atherosclerosis during inflammaging^62,63^. Pro-inflammatory cytokines impair ECs and BBB function, and induce the expression of adhesion molecules and chemokines recruiting leucocytes to cerebral lesions. In the spontaneously hypertensive rat (SHR), peripheral blood and infiltrating immune cells (e.g., T cells, NK cells) were found to expand in the cerebral blood vessels, leading to inflammation, endothelial dysfunction, and ischemia in this area^64^. The increased expression of adhesion molecules might lead to an increased number of T cells in SHR brains. On one hand, T cells adhering to the lumen side of brain microvessels might be part of the systemic adaptive immune response to angiogenesis antigens during hypertension. On the other hand, T cells directly promoted endothelial dysfunction, and might impair brain perfusion by microvascular plugging and thrombosis^65^. In addition, a variety of anti-endothelial cell antibodies were found in the serum of CSVD patients, suggesting a possible mechanism that B cell activation promotes endothelial dysfunction^66^. During inflammaging, monocytes activate pathways that promote inflammatory polarization, driving ROS production and hypertension^67^. Macrophages infiltrating vascular walls produce elevated levels of ROS, reduce the use of NO, promote the expression of adhesion molecules, stimulate vascular smooth muscle cell (VSMC) hypertrophy, and activate matrix metalloproteinases, all of which are involved in vascular remodeling and dysfunction^68^.

Inflammasomes are multiprotein signaling complexes. They play a key role in the mediation of innate inflammatory responses and are assembled in response to a wide range of stimuli including both PAMPs and DAMPs^69^. Although several types of inflammasomes have been identified so far, the best characterized is the NOD-like receptor family pyrin domain containing 3 (NLRP3) inflammasome^70^. The activation of NLRP3 inflammasome requires two steps to strictly regulate its functions. First, the initiation step increases the expression of individual inflammasome components and assembles them into macromolecular multimers. Then, various potentially harmful stimuli provide a second signal that endows the inflammasome component caspase-1 with activity. This proteinase cleaves the inactive precursors of the pro-inflammatory cytokines such as IL-1β and IL-18, conferring them biological activity^71^. During inflammaging, the continuous increase of inflammatory mediators and the decrease of anti-inflammatory cytokines (e.g., IL-10, adiponectin) may exacerbate vascular extracellular matrix remodeling and arterial stiffening, thereby accelerating plaque formation^72^.

NLRP3 inflammasome is recognized as an important mediator linking atherosclerosis and inflammaging^73^. Atherosclerotic lesions produce DAMPs (e.g., cholesterol crystals) that activate NLRP3 inflammasomes in macrophages and generate active forms of pro-inflammatory cytokines (such as IL-1β and IL-18)^67^. The resulting inflammatory environment leads to the formation of complex plaques by mediating cellular recruitment and the generation of foam cells and fatty streaks. Besides, IL-1β and IL-18,as the main products of NLRP3 activation, trigger the expression of IL-6 in many types of cells^74^. This cascade reaction greatly amplifies the inflammasome signal. IL-6 can promote thrombotic events by producing of fibrinogen and plasminogen activator inhibitor-1 in the liver, thereby making the blood more coagulable and impairing fibrinolysis^75^.

Accumulated SCs are detected in advanced atherosclerotic plaques by measuring specific markers such as senescence-related β galactosidase (SAβG), p16, and tumor suppressor replacement reading frame (ARF). Compared with age-matched healthy vessels, the proportion of SAβG-positive cells in the intimal and medial layers of atherosclerosis increase^76,77^. SCs contribute to the development of inflammaging through SASP^78^. Wang et al.^79^ found that the increase of matrix metalloproteinases enhances the decomposition of the extracellular matrix and promotes the reconstruction of advanced atherosclerotic plaques. The decomposition of the extracellular matrix promotes the migration of VSMC from media, mediates the compensatory enlargement of arteries, and weakens the protective fibrous cap of plaques^80^.

Metaflammation and gut microbiota dysbiosis play an important role in arterial hypertension and vascular dysfunction induced by inflammaging. Overweight and obese patients tend to suffer from a state of metaflammation. In the post-prandial period, their circulating VLDL could induce the expression of adhesion molecules, cytokines, and pro-oxidants in ECs and leucocytes, which is conducive to the occurrence of vascular inflammation^81,82^. Moreover, insulin resistance mediated by metabolic inflammation is a co-recognized vascular risk factor associated with aging. In this way, inflammaging amplifies the vicious cycle of obesity, insulin resistance, aging, and vascular dysfunction^83^. Besides, aging increases the accumulation of monocytes and lymphocytes in adipose tissue^84^, which are considered as critical mediators of inflammation and oxidative stress in arterial hypertension.

Research on cardiovascular (CV) disease have reported the presence of bacteria in atherosclerotic plaques^85,86^, suggesting that gut microbiota may influence age-related CV inflammation. In our recent study, we found that the altered microflora also affected the pathophysiology of atherosclerotic CSVD (aCSVD). Besides, we evaluated the inflammatory status of aCSVD patients by quantifying the mRNA levels of inflammaging markers in circulating leukocytes. The expression levels of the inflammaging markers (including IL-17A, TNF-α, IL-6, and IFNα) increased in aCSVD patients compared with the healthy control. The tested inflammaging markers displayed prognostic significance, expression of IL-1β, IL-6, IFN-α, and IL-17A increased the predicting sensitivity of detrimental imageology signs (unpublished data).

### Systemic inflammation and CAA

Sporadic (non-inflammatory) CAA is specifically characterized by the progressive deposition of amyloid β40 (Aβ40) protein (to a lesser extent, Aβ42): (i) capillary CAA (CAA-type 1), which mainly affect the walls of capillaries and the surrounding neuropilcapillary CAA (CAA-type 1), (ii) arterioles and rarely veins (CAA-type 2), which mainly involve noncapillary blood vessels such as small to medium-sized arteries of parenchymal and leptomeningeal^87^. Regional studies shown that systemic inflammation was preferentially associated with vascular injury in areas commonly associated with CAA pathology (lobular regions supplied by cortical and leptomeningeal vessels)^6^.

Aβ is derived from the enzymatic cleavage of transmembrane amyloid precursor proteins in cells (e.g., neurons and oligodendrocytes). Soluble Aβ can be removed from the brain by various clearance systems, including enzymatic degradation and cellular uptake, transport across the BBB, interstitial fluid (ISF) bulk flow, and cerebrospinal fluid (CSF) absorption into the circulatory and lymphatic systems^88,89^. In addition, mechanisms eliminating Aβ out of the brain depend on the integrity of cerebral microvessels, whereas the risk of vascular wall damage increases with age.

As sentinels of the brain, microglia exhibit multiple responses to external stimuli and play an important role in Aβ clearance. During aging, microglia acquire an activated phenotype, morphologically characterized by reduced branches and increased cell soma volume; functionally defined by the release of pro-inflammatory cytokines such as IL-1β, TNF-α, and IL-6^90–92^. The inflammatory responses initiated by microglia involves multiple inflammasomes, among which NLRP3 inflammasome is the most characteristic and most widely implicated regulators of IL-1β and IL-18^93,94^. In a murine model assessing microglial dynamics in the context of systemic inflammation, 3-dimensional and quantitative analyses of microglia revealed that systemic inflammation (i) briefly affected microglia in an age-dependent manner; (ii) increased Aβ deposition by affecting microglial cell clearance; (iii) increased microglial cell proliferation and served as a marker of disease acceleration. Moreover, the activation of NLRP3 inflammasome could be identified as a vital mediator of these effects^95^.

Chen Y et al.^96^ found that NLRP3 activation could impair endothelial permeability and barrier function under stress conditions such as hyperlipidemia. In high-fat diet-fed mice, the harmful adipose factor visfatin increased in the serum, which acted on the ECs, activated NLRP3 to promote the release of downstream high mobility group box-1 protein 1 (HMGB1), and further caused a downregulation of interendothelial connexin ZO-1, ZO-2, occludin, and VE-cadherin, leading to increased endothelial permeability and impaired barrier function. Moreover, elevated oxidative stress and defective autophagy also activate NLRP3 inflammasome in senescent endothelium^97^. This process further aggravates vascular endothelial dysfunction, compromises Aβ clearance and promotes the occurrence and development of CAA.

In addition to innate immune cells, the response of adaptive immune cells, especially T cells, to brain amyloidosis has also received increasing attention. In transgenic (tg) mouse models of AD-like cerebral amyloidosis, studies shown that brain amyloidosis promoted T cell infiltration but interfered with local antigen presentation and T cell activation. In the long term, the ability of microglia and macrophages to remove Aβ might be reduced in the absence of T cell-derived stimulants and/or in response to T cell-derived inhibitory signals^98^.

The brain-gut axis refers to the bidirectional, continuous communication between the central nervous system and the gastrointestinal tract^99^. The term has recently been extended to the brain-gut-microbiota axis. There is increasing evidence that gut microbiota affects brain-gut interactions at different time points (from early life to aging) and at different levels (from the lumen to the central nervous system). Gut microbiota provides two-way communication through immune, hormone, and neuronal signals^100^. Inflammaging causes alterations in gut microbiota composition and decreases in microbiota diversity and stability, resulting in the destruction of the intestinal barrier. Disrupted intestinal barrier further increase circulating pro-inflammatory cytokines and bacterial derivatives, triggering systemic inflammation and BBB damage^101^.

Gut microbiota may affect the function of the central nervous system by directly synthesizing various neurotransmitters and neuromodulators. For example, signals from the gut microbiome may regulate the function of intestinal enterochromaffin cells, which produce different hormones and neurotransmitters, such as serotonin^102^. Moreover, the results of studies in germ-free mice confirmed that microbiota may be mediated by bacterial metabolites short-chain fatty acids (SCFAs), thereby affect microglia maturation and reduce BBB permeability^100^.

**Fig. 3:**
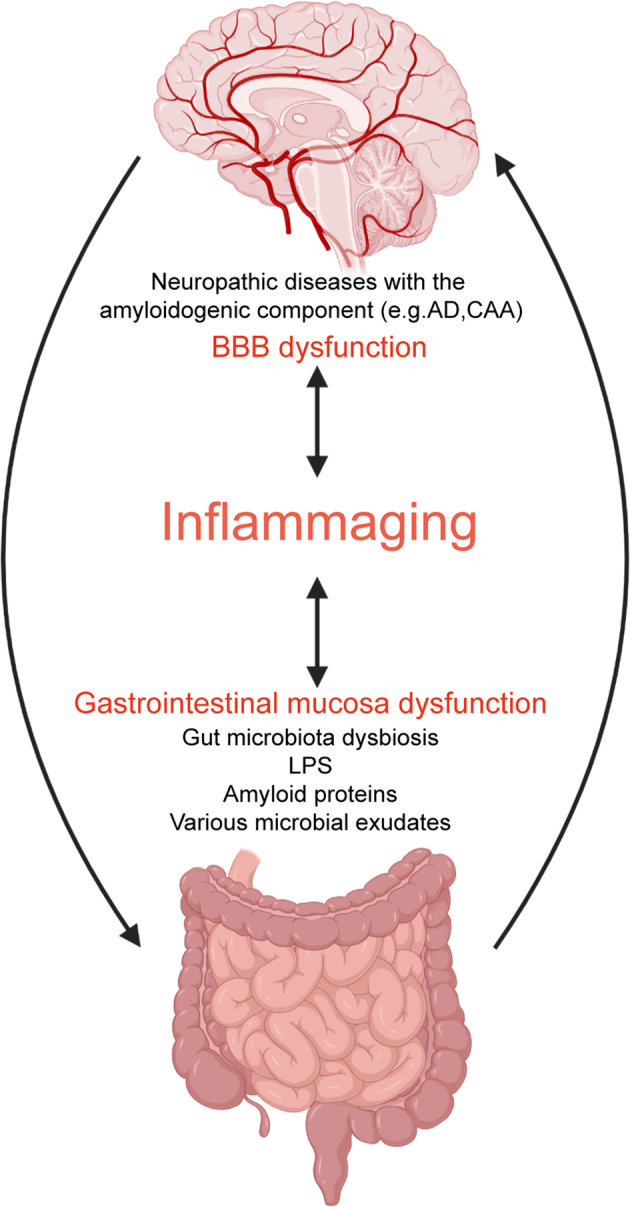
Pathogenic role of the brain-gut-microbiota axis dysfunction during inflammaging. In the process of inflammaging, gastrointestinal mucosa and blood-brain barrier (BBB) function are impaired, and the brain-gut-microbiota axis function is disturbed. Pathogenic microbiota and their products (LPS, amyloid proteins, and various microbial exudates) can further induce inflammatory responses and dysfunction of the brain-gut-microbiota axis, ultimately leading to the neuropathic diseases with the amyloidogenic component.

The diverse gut microbiota produce large amounts of lipopolysaccharide (LPS), amyloid proteins, and various microbial exudates^103–105^. Microbial and cerebral amyloids are biologically similar in higher-order structure, PAMPs composition, and physic-chemical characteristics, despite of their difference from the human Aβ1–42 amino acid sequences^104^. In addition, they are also recognized by the same TLR2 / TLR1 receptor system as Aβ42, which strongly activates the production of pro-inflammatory cytokines, particularly IL-17 and IL-22. In the innate immune system, LPS activates TLRs expressed in microglia cells, which recognize common injuries or PAMPs^106^. LPS activates the TLR4 receptor and promotes inflammation by interacting with CD14 and MD-2 proteins. The activation of TLR4 in CD14 also mediates the inflammatory response to Aβ^107^. Secretion from the total microbiome constitute a very powerful class of pro-inflammatory complement and innate immune activators. They possess great potential for inducing pro-inflammatory cytokines, complement activation and altering immunogenicity in the brain. Both amyloid proteins and LPS are potent activators of the receptors for advanced glycosylation end products (RAGE) and TLRs. This pathogenic effect enhances amyloid accumulation and increases the inflammatory response^108^. In daily life, people are also exposed to large amounts of LPS and amyloid proteins continuously produced by the gut microbiota. Such exposure may be harmful to health. This deleterious effect is more evident when the gastrointestinal mucosa and BBB are remodeled during inflammaging (Fig. 3)^104,105^. However, current studies on gut microbiota of neuropathic diseases with the amyloidogenic component are mostly focused on Alzheimer’s disease, with no animal or clinical research on CAA.

DPA and CAA are extreme outcomes of the pathologic continuum of age-related CSVD. Endothelial injury, destruction of the BBB and impaired perivascular Aβ drainage are the main mechanisms of age-related CSVD^2^. A comprehensive and in-depth understanding of the relationship between inflammaging and age-related CSVD will help us propose targeted anti-CSVD treatment plans.

## Possible therapeutic interventions

Treatment of age-related CSVD includes specific and non-specific treatment. Non-specific treatment includes controlling vascular risk factors, antiplatelet therapy, intravenous thrombolysis, etc.^1^. In particular, antiplatelet and thrombolytic therapy reduces the risk of ischemic stroke but increases the risk of cerebral hemorrhage as well. Thus, it is necessary to balance the relationship between these two aspects to ensure the maximum benefit of treatment^109^.

With the deepening understanding of the pathogenesis of age-related CSVD, new therapeutic interventions have been proposed (Table 1). Recently, specific therapeutic targets for inflammaging have attracted increasing attention. Shuzhen Z et al.^110^ proposed that lipoprotein-associated phospholipase A2 (Lp-PLA2) and superoxide dismutase (SOD), were independently related to cognitive dysfunction and WMH lesion of CSVD, and could be considered as therapeutic targets to prevent age-related CSVD. Several anti-inflammatory drugs, such as fingolimod, natalizumab, dimethyl fumarate, and rituximab, have been applied to treat neuroinflammatory diseases such as multiple sclerosis^54^. Ongoing studies are evaluating the effectiveness of anti-inflammatory drugs for CSVD, but clinical evidence is still lacking^109^.

**Table 1:** New progress in therapeutic interventions of age-related CSVD.

Targets	Mechanisms of action	Intervention Strategies	References
Vascular/neuro-inflammation	-Regulate the differentiation and migration of lymphocytes -Impact the anti-oxidative stress cell machinery	-Anti-inflammatory drugs **·**Fingolimod **·**Natalizumab **·**Dimethyl fumarate **·**Rituximab	^54,109,110^
Integrity of the BBB	-Maintain the stability of endothelial function -Promote interactions between pericytes and ECs	-EC-stabilizing drugs **·**Statins **·**ACEIs **·**Cilostazol	^111–116^
Stem cell	-Remodel of microvasculature through MSCs -Reprogram of pericytes to promote vasculogenesis	-Intravenous infusion of MSCs -Intracranial pericytes implantation	^117–122^

An important pathophysiological mechanism of CSVD, endothelium dysfunction and subsequent BBB leakage, provides an option for the targeted therapy of CSVD. In rat models of CSVD, ECs dysfunction was found to be the first change in the development of CSVD, and treatment with EC-stabilizing drugs (e.g., statins, ACEIs, cilostazol) could reverse these EC and oligodendroglial pathologies^111^. In clinical trials, statins have previously been shown to reduce stroke rates in CSVD patients, but only in patients with elevated cholesterol^112^. Trials using ACEIs alone, excluding other types of antihypertensive agents, have shown a more active role in preventing WMH progression in CSVD^113^. Cilostazol is currently undergoing early stage clinical trials for CSVD. In addition, pericyte is an under-studied cell type that wraps the ECs and is embedded in the basement membrane outside brain vessels including capillaries, post-capillary venules, and terminal arterioles^114^. Pericytes play a vital role in regulating various microvascular functions, such as angiogenesis, BBB preservation, capillary blood flow, and the migration of immune cells into the brain. Molecules mediating pericyte–EC interactions, such as platelet-derived growth factor-BB (PDGF-BB), TGF-β, have been proposed as targets for the treatment of neurological disorders^115^. In a cell culture model, using PDGF-BB or TGF-β could better maintain BBB function under hypoxia conditions^116^. However, in CSVD, animal and clinical trials of relevant drugs have not yet been conducted.

Stem cell therapy has attracted increasing attention in recent years. Intravenous infusion of MSCs have been shown to inhibit the deterioration of BBB function and improves the functional outcome in a middle cerebral artery occlusion (MCAO) model^117^. In a CSVD model SHR (stroke-prone) with impaired cognitive, the infusion of MSCs was found to exert a therapeutic effect on CSVD. It could restore the BBB function via remodeling of microvasculature and reduce the Aβ accumulation, by which inhibiting progressive brain atrophy and restoring cognitive dysfunction^118^. Therefore, the intravenous administration of MSCs would be a new and reasonable therapeutic strategy for age-related CSVD. Moreover, a growing body of evidence indicates that pericytes are multipotential stem cells^119^. Recent studies have demonstrated that pericytes are capable to differentiate into neurons, microglia, and vasculature after brain injuries in ischemic disease and hypoxia conditions^120,121^. In amyloid model mice, pericyte implantation in the brain increased cerebral blood flow and reduced pathological deposition of Aβ^122^. These findings suggest that transplantation of pericytes may be a promising approach to treat age-related CSVD.

## Future directions

Although considered to be independent lesions in the past, studies have suggested that DPA and CAA interact and overlap in pathological mechanisms, which can be regarded as the extreme outcomes of the age-related CSVD development^2^. Given the above-mentioned effects of inflammaging on systemic and vascular inflammation, it may help to explain the pathophysiological interactions between DPA and CAA.

Gut microbiota dysbiosis is an essential link between inflammaging and CSVD. However, most of the current studies focus on the relationship between gut microbiota and neurodegenerative diseases (e.g., Alzheimer’s disease). Future research should focus on the co-relationship between gut microbiota of age-related CSVD patients and inflammaging.

At present, therapeutic interventions of age-related CSVD targeted for inflammaging, endothelium dysfunction, and subsequent BBB leakage, have been proposed, but most of them only enter the molecular and animal experimental stage. In the future, more clinical trials should be conducted to evaluate their safety and effectiveness.

## Conclusion

Aged individuals commonly undergo a chronic sterile low-grade inflammation called inflammaging, in which a variety of cellular and biological mechanisms play a role. In the elderly, inflammaging predicts the risk of various age-related chronic non-infectious diseases, including age-related CSVD. There are two main types of age-related CSVD, namely DPA and CAA. Regional analyses show that systemic and vascular inflammation have correlations with two distinct types of CSVD respectively. The close relationship between inflammaging and age-related CSVD suggests the potential for inflammaging-related therapy. However, few studies have focused on inflammaging-related therapies and their effects on age-related CSVD. This gap is an important area of unmet medical need that deserves further translational research.
